# Nanofibrous Conductive Binders Based on DNA-Wrapped Carbon Nanotubes for Lithium Battery Electrodes

**DOI:** 10.1016/j.isci.2020.101739

**Published:** 2020-10-28

**Authors:** Ju-Myung Kim, Seung-Hyeok Kim, Nag Young Kim, Myeong-Hwa Ryou, Hongyeul Bae, Jin Hong Kim, Young-Gi Lee, Sang-Young Lee

**Affiliations:** 1 Department of Energy Engineering, School of Energy and Chemical Engineering, Ulsan National Institute of Science and Technology (UNIST), Ulsan 44919, South Korea; 2 Energy Materials Research Group, Research Institute of Industrial Science and Technology (RIST), Pohang-si, Gyeongsangbuk-do 37673, South Korea; 3 Intelligent Sensors Research Section, ICT Creative Research Laboratory, Electronics and Telecommunications Research Institute (ETRI), Daejeon 34129, South Korea; 4 Department of Chemical and Biomolecular Engineering, Yonsei University, 50 Yonsei-ro, Seodaemun-gu, Seoul 120-749, South Korea

**Keywords:** Nanotechnology, Nanomaterials, Energy Materials

## Abstract

In contrast to enormous progresses in electrode active materials, little attention has been paid to electrode sheets despite their crucial influence on practical battery performances. Here, as a facile strategy to address this issue, we demonstrate nanofibrous conductive electrode binders based on deoxyribonucleic acid (DNA)-wrapped single-walled carbon nanotubes (SWCNT) (denoted as DNA@SWCNT). DNA@SWCNT binder allows the removal of conventional polymeric binders and carbon powder additives in electrodes. As a proof of concept, high-capacity overlithiated layered oxide (OLO) is chosen as a model electrode active material. Driven by nanofibrous structure and DNA-mediated chemical functionalities, the DNA@SWCNT binder enables improvements in the redox reaction kinetics, adhesion with metallic foil current collectors, and chelation of heavy metal ions dissolved from OLO. The resulting OLO cathode exhibits a fast charging capability (relative capacity ratio after 15 min [versus 10 h] of charging = 83%), long cyclability (capacity retention = 98% after 700 cycles), and thermal stability.

## Highlights

• A multifunctional binder based on DNA-wrapped SWCNT is demonstrated
• DNA@SWCNT binder shows improved adhesion with metallic foil current collectors
• DNA@SWCNT binder enables chelation of heavy metal ions dissolved from cathode materials
• DNA@SWCNT binder-containing cathode exhibits fast-charging capability

## Graphical Abstract

**Figure:**
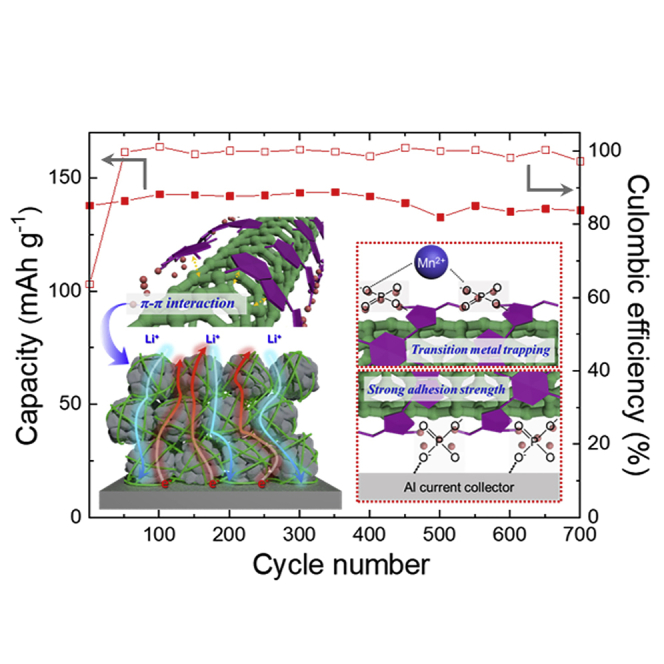

## Introduction

Despite their successful commercialization in various application fields, lithium-ion batteries (LIBs) have still faced challenges in terms of the energy density, fast charging, long-term sustainability, and safety (Liu et al., 2019; Schmuch et al., 2018). Previous works implemented to resolve these issues have mostly focused on the synthesis and modification of electrode active materials (Zeng et al., 2018; Nayak et al., 2018; Ji, 2019; Lu et al., 2016). Meanwhile, considering the fact that a practical cell performance is eventually governed by electrode sheets, significant attention should be undoubtedly devoted to electrode sheet design. Conventional electrode sheets consist of electrode active materials, polymeric binders, and carbon conductive powders on top of metallic foil current collectors. This electrode architecture has limitations in providing well-interconnected ion/electron pathways that are essentially secured for reliable redox reactions, which become more serious in the high-mass loading/high-thickness electrodes that are recently spotlighted for realizing high-energy density batteries (Tang et al., 2015; Wang et al., 2019). Moreover, the insufficient adhesion between the electrode components and current collectors is another concern in electrode sheet development (Yuan et al., 2018; Bresser et al., 2018).

Several approaches have been explored to address the aforementioned issues, with a focus on facilitating the electron/ion transport phenomena in the electrode sheets by (1) incorporation of conducting polymers, such as polypyrrole (Shi et al., 2017) and poly3,4-propylenedioxythiophene-2,5-dicarboxylic acid (Ling et al., 2015), (2) use of nickel (Ni)-supported, alumina (Al_2_O_3_)-coated three-dimensional (3D)-structured scaffolds (Kim et al., 2017a), and (3) introduction of lithium polyacrylic acid (Pieczonka et al., 2015; Su et al., 2018) as an extra lithium ion source. In addition, new binder materials based on hydrogen bonding, including styrene-butadiene rubber/carboxymethylcellulose (Yabuuchi et al., 2015), xanthan gum (Zhang et al., 2019), and polyacrylic latex (Zhong et al., 2016), have been investigated to achieve strong adhesion with metallic current collectors. Unfortunately, most of these previous works have not yet reached a satisfactory electrochemical performance and still relied on complex, expensive, and time-consuming synthesis/manufacturing processes.

Here, intrigued by the chemical/structural features of deoxyribonucleic acid (DNA) molecules and carbon nanotubes, we demonstrate a new class of nanofibrous conductive electrode binders based on DNA-wrapped single-walled carbon nanotubes (SWCNTs) (referred to as DNA@SWCNT). Note that electrode active materials are spatially surrounded by highly reticulated DNA@SWCNT networks, which allows the removal of traditional polymeric binders and carbon powder additives in electrodes. Among several CNT candidates, we choose SWCNT owing to its high electronic conductivity and large aspect ratio, which are beneficial for forming well-interconnected network structure in the electrode sheets.

DNA is an amphiphilic molecule composed of hydrophobic aromatic base pairs and hydrophilic phosphate backbones (Guo et al., 2012; Chen et al., 2007). The hydrophobic aromatic bases of DNA can intimately interact with the CNT through noncovalent π–π stacking interactions (Kim et al., 2020; Zheng et al., 2003), thus enabling the preferential presence of the hydrophilic phosphate groups of DNA in the outmost layers of the DNA@SWCNT. These spatially rearranged phosphate groups of DNA are expected to play a viable role in achieving strong adhesion with aluminum (Al) current collectors and chelating the heavy transition metal ions (e.g., manganese [Mn^2+^] ions) that are dissolved from cathode active materials. Furthermore, the phosphate groups, owing to their hydrophilicity, enable aqueous solution-based eco-friendly electrode fabrication processes instead of typical N-methyl pyrrolidone (NMP) solvent-based electrode preparation processes. In addition to these DNA-driven chemical functionalities, the nanofibrous structure of the DNA@SWCNT binder allows liquid electrolytes to easily access the electrode active materials. As a result, 3D bicontinuous ion/electron conduction pathways are formed in the electrode sheets.

As a proof of concept for the DNA@SWCNT binder, we chose an overlithiated layered oxide (OLO, 0.49Li_2_MnO_3_·0.51LiNi_0.37_Co_0.24_Mn_0.39_O_2_), which has garnered considerable attention as a next-generation high-capacity (∼250 mAh g^−1^) (Zhou et al., 2018) cathode material. However, OLO is still far from practical use because of some unresolved problems (Mun et al., 2014; Zhan et al., 2018; Chung et al., 2002; Nayak et al., 2016), such as its low electronic conductivity (10^−9^ S cm^−1^), interfacial side reactions with liquid electrolytes, and accompanying dissolution of the transition metal ions, resulting in a poor electrochemical performance and structural instability.

Benefiting from the advantageous effects described above, the DNA@SWCNT binder provides improvements in the redox kinetics, adhesion with Al current collectors, and heavy metal ion chelation. Consequently, the resulting OLO cathode (D@S−OLO cathode) exhibits a fast charging capability (the relative capacity ratio after 15 min [versus 10 h] of charging = 83%), long cyclability (capacity retention = 98% after 700 cycles), and thermal stability. To the best of our knowledge, the DNA@SWCNT-based nanofibrous conductive binder strategy proposed herein has not been reported in battery electrodes.

## Results and Discussion

### Characterizations of DNA-Wrapped SWCNT

**Figure 1:**
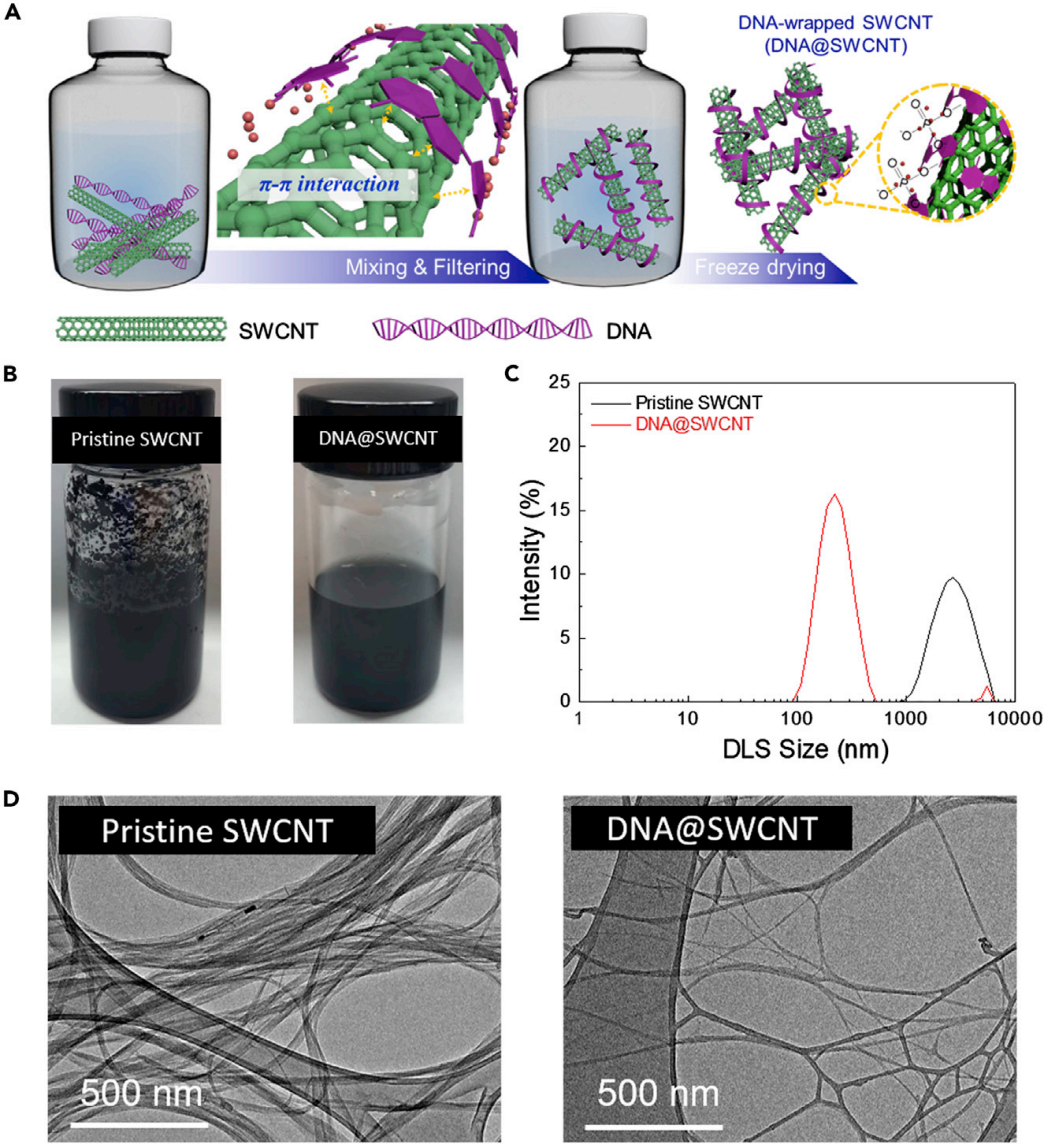
Preparation and Characterization of the DNA-Wrapped SWCNT (DNA@SWCNT) (A) Schematic illustration depicting the stepwise preparation of the DNA@SWCNT. (B) Photographs of the aqueous suspensions: pristine SWCNT (left) and DNA@SWCNT (right). (C) DLS number-distribution of the aqueous suspensions (pristine SWCNT versus DNA@SWCNT). (D) TEM images of the aqueous suspensions: pristine SWCNT (left) and DNA@SWCNT (right).

The stepwise process used to prepare the DNA@SWCNT is schematically depicted in Figure 1A. The DNA@SWCNT aqueous suspension (with an initial composition ratio of DNA/SWCNT = 7/3 (w/w)) was prepared by simple mixing and subsequently by filtering. Figure 1B compares the dispersion state of the DNA@SWCNT with that of the pristine SWCNT in the aqueous suspensions. The DNA@SWCNT, owing to the conformal wrapping of the SWCNT by DNA via intermolecular π–π stacking interactions (Kim et al., 2020; Zheng et al., 2003), showed a better dispersion state than the pristine SWCNT. This result was verified by dynamic light scattering (DLS) analysis (Figure 1C). The pristine SWCNT suspension showed severe aggregation, with an average hydrodynamic particle diameter of ∼2,670 nm, which appeared to be similar to those (Vidali et al., 2016) of previous studies. In comparison, the particle diameter was remarkably reduced to ∼220 nm for the DNA@SWCNT suspension. Such a good dispersion state enabled by DNA was further confirmed by transmission electron microscopy (TEM) (Figure 1D). The SWCNT fibrils of the DNA@SWCNT suspension were better dispersed than those of the pristine SWCNT suspension, demonstrating the advantageous effect of DNA on the SWCNT dispersion. The above-prepared DNA@SWCNT suspension was subjected to freeze-drying, yielding DNA@SWCNT powders. The composition ratio of the obtained DNA@SWCNT powders was estimated to be 76/24 (w/w) from the elemental analysis (Figure S1).

### Electrochemical/Physical Properties of the DNA@SWCNT

**Figure 2:**
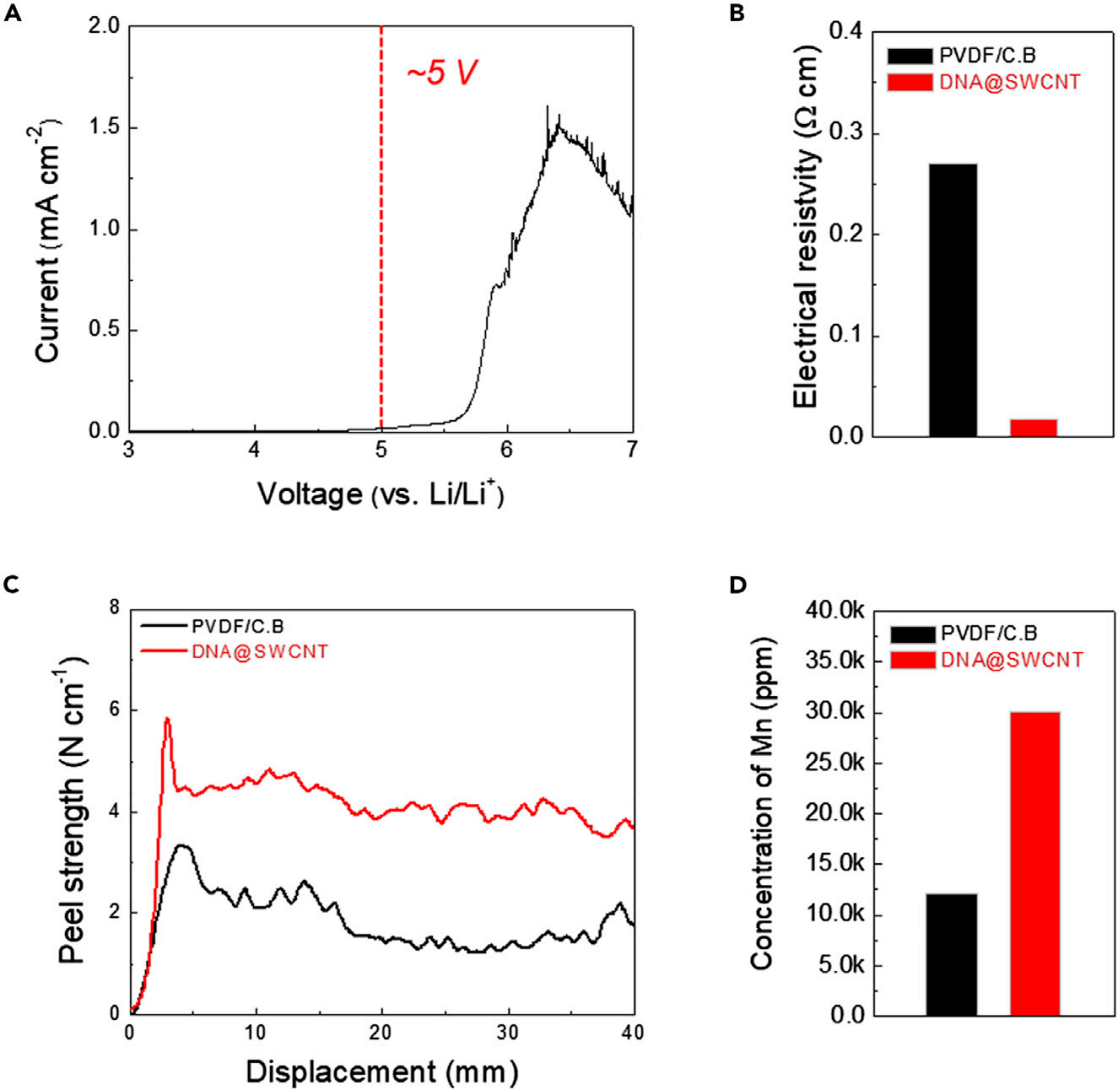
Electrochemical/Physical Characterization of the DNA@SWCNT Film (A) Electrochemical stability window of the DNA@SWCNT film. (B) Comparison of the surface electrical resistivity: DNA@SWCNT film versus control (PVdF/carbon black) film. (C) The 180° peel-off test, showing the adhesion between the DNA@SWCNT film (versus the control film) and Al current collector. (D) Comparison of the amount of Mn^2+^ ions trapped by the DNA@SWCNT film versus the control film (estimated by the ICP-MS analysis).

The major electrochemical/physical properties of the DNA@SWCNT were investigated to explore its potential for use as an electrode binder. As a supplementary experiment, a film made of DNA@SWCNT was fabricated and compared with a control film (comprising a mixture with a polyvinylidene fluoride [PVdF]/carbon black powder ratio = 5/5 (w/w)). The linear sweep voltammetry (LSV) profile (Figure 2A) showed that the DNA@SWCNT film is electrochemically stable up to ∼5 V (versus Li/Li^+^), which is superior to that (Figure S2) of the control film. We measured the surface electrical resistivity of the DNA@SWCNT film (Figure 2B). Despite the smaller content of conductive carbon, the DNA@SWCNT film exhibited a lower surface electrical resistivity (0.02 Ω cm) than the control film (0.27 Ω cm), revealing the formation of well-developed electronic conduction pathways in the DNA@SWCNT film. The adhesion between cathode active layers and Al current collectors critically affects the overall electrochemical performance of cathode sheets. The DNA@SWCNT and control films were laminated with Al current collectors and then subjected to a 180° peel-off test. The DNA@SWCNT film showed a higher adhesion strength than the control film (Figure 2C), indicating the advantageous effect of DNA (specifically, phosphate groups) compared with the conventional PVdF binder.

We investigated the effect of the composition ratio (DNA/SWCNT) on the electrical resistivity and adhesion strength of the DNA@SWCNT films. The other initial composition ratios of the DNA/SWCNT (3/7 and 5/5 (w/w)) showed a lower adhesion strength than the above-mentioned initial composition ratio of the DNA/SWCNT (7/3 (w/w)), whereas the enhancement in the electrical resistivity was not so noticeable (Figure S3). To provide an additional evidence, OLO cathodes with different composition ratios of DNA/SWCNT were prepared as control samples and their electrochemical performance was compared. The OLO cathode (DNA/SWCNT = 7/3 (w/w)) showed the superior cycling performance (at a charge/discharge current density of 1C/1C) compared with the OLO cathode (DNA/SWCNT = 5/5 (w/w)) (Figure S4). Based on these results, the optimal initial composition ratio of the DNA/SWCNT was determined to be 7/3 (w/w).

A formidable challenge of high-capacity/high-voltage cathode materials is the dissolution of heavy metal ions into the electrolytes, eventually causing a decline in the cell performance during charge/discharge cycling (Zhan et al., 2018). To examine the Mn^2+^ chelating ability, the DNA@SWCNT film was immersed for 2 h in a model liquid electrolyte (a manganese perchlorate [Mn(ClO_4_)_2_] electrolyte solution) (Kim et al., 2015) (i.e., 10 mM Mn(ClO_4_)_2_-containing 1.0 M LiPF_6_ in ethylene carbonate [EC]/dimethyl carbonate [DMC] = 1/1 (v/v)), and then, the trapped Mn^2+^ was quantitatively estimated using inductively coupled plasma mass spectrometry (ICP-MS). Figure 2D shows that the amount of Mn^2+^ trapped by the DNA@SWCNT film (30,120 ppm) is substantially higher than that trapped by the control film (12,059 ppm), verifying the viable role of the DNA phosphate groups in chelating the heavy metal ions.

### Structural/Electrochemical Characterization of the D@S−OLO Cathode

**Figure 3:**
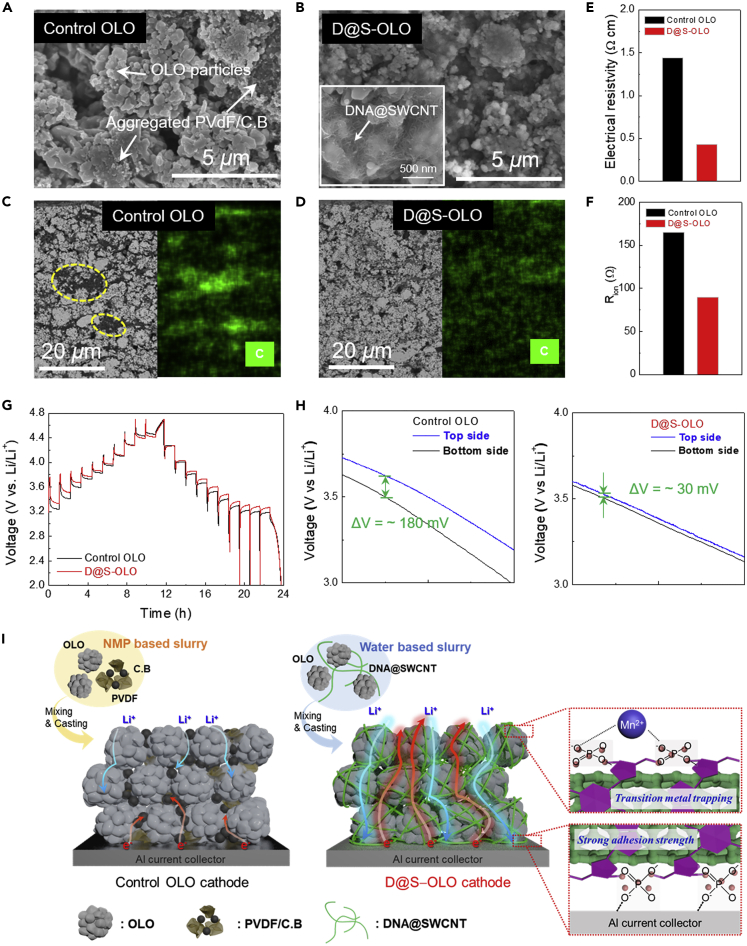
Structural/Electrochemical Characterization of the D@S−OLO Cathode Surface SEM images of (A) the control OLO cathode and (B) D@S−OLO cathode. Cross-sectional SEM images with the carbon (represented by green dots) EDS mapping images of (C) the control OLO cathode and (D) D@S−OLO cathode. (E) Comparison of the surface electric resistivity: D@S−OLO cathode versus control OLO cathode. (F) Comparison of the ionic resistance (R_ion_) at 50% SOC: D@S−OLO cathode versus control OLO cathode. (G) GITT profiles obtained upon the repeated current stimuli (at a current density = 1 C, where the interruption time between the pulses = 1 h). (H) Overpotential distribution of the cathodes (control OLO cathode [left] versus D@S−OLO cathode [right]) in the through-thickness direction (i.e., voltage difference between the top and bottom side of the cathodes) at charge/discharge current densities of 1 C/1 C. (I) Schematic illustration showing the structural difference between the control OLO and D@S−OLO cathodes. The advantageous effect of the D@S−OLO cathode on the electron/ion transport pathways, adhesion with an Al current collector, and Mn^2+^ ion chelation is conceptually depicted.

An OLO cathode incorporating the DNA@SWCNT binder was fabricated by casting an aqueous OLO slurry (OLO/DNA@SWCNT = 92/8 (w/w)) onto an Al current collector. The obtained OLO cathode (referred to as the D@S−OLO cathode) showed an areal mass loading of ∼7 mg cm^−2^ and a thickness of ∼40 μm. A conventional OLO cathode (OLO/(PVdF/carbon black) = 92/(4/4) (w/w/w)) with a similar areal mass loading (∼7 mg cm^−2^) and thickness (∼39 μm) was prepared as a control sample. The obtained D@S−OLO cathode showed thermal stable behavior up to 800°C in the thermal gravimetric analysis (TGA) under inert gas (Figure S5). The surface scanning electron microscopy (SEM) image (Figure 3A) showed that the OLO particles were not homogeneously dispersed in the control OLO cathode, along with some aggregates of PVdF/carbon black mixtures. In comparison, the OLO particles spatially surrounded by the DNA@SWCNT binders were uniformly dispersed in the D@S−OLO cathode (Figure 3B) owing to the DNA phosphate groups, which can form hydrogen bonds with metal oxides (Guo et al., 2012). Such a morphological difference between the two cathodes was verified by analyzing the cross-sectional SEM and energy dispersive X-ray spectroscopy (EDS) images (Figures 3C, 3D, and S6). Consistent with the results shown in Figure 3A, the control OLO cathode showed a nonuniform and aggregated dispersion state (denoted by the yellow circles in the SEM image and green dots [representing carbon (C)] in the EDS image). In contrast, a uniform/homogeneous dispersion state was observed in the D@S−OLO cathode. This result was further confirmed by comparing the surface electrical resistivity of the OLO cathodes (Figure 3E). The D@S−OLO cathode exhibits a lower electrical resistivity (0.43 Ω cm) than the control OLO cathode (1.44 Ω cm). In addition, we measured the interfacial electrical resistance between the cathode active layers and Al current collectors (Figure S7). The D@S−OLO cathode showed a considerably lower interfacial contact resistance (0.1 Ω cm^−2^) than the control OLO cathode (0.66 Ω cm^−2^), exhibiting the intimate electrical contact between the D@S−OLO cathode and the Al current collector. On the other hand, the OLO cathode with multiwalled carbon nanotube (MWCNT) in which the same composition ratio (OLO/DNA@MWCNT = 92/8 (w/w), DNA/MWCNT = 7/3 (w/w)) as D@S−OLO cathode exhibited higher electrical resistivity of 1.18 Ω cm than the D@S−OLO cathode (Figure S8). This result demonstrates the superiority of the SWCNT compared with MWCNT in forming the well-developed electrical channels, mainly owing to its highly reticulated fibrous structure.

The interfacial adhesion with the Al current collector was examined in more detail. A peel-off test was conducted for the D@S−OLO cathode after it was soaked in the liquid electrolyte to explore its applicability in practical cells. The excellent structural robustness of the D@S−OLO cathode was observed (Figure S9A) and compared with that of the control OLO cathode, showing the severe detachment of its components (Figure S9B). As additional evidence, we measured the contact resistance between the OLO cathodes (taken after the peel-off test) and Li metals. The D@S−OLO cathode had a higher resistance than the control cathode owing to the less exposed area of the Al current collector (Figures S9C and S9D), which may mitigate catastrophic cell failure upon the occurrence of an internal short circuit. This result exhibits the superior structural stability of the D@S−OLO cathode against external mechanical stress.

The effect of the DNA@SWCNT binder on the ion transport phenomena of the D@S−OLO cathode was investigated by conducting electrochemical impedance spectroscopy (EIS) for a symmetric cell (comprising two identical cathodes at a state of charge [SOC] of 50%). The obtained Nyquist plots can deliver information on the cathode-electrolyte interface (Sun et al., 2017; Landesfeind et al., 2016). Both the Nyquist plots obtained for the D@S−OLO and control OLO cathodes showed 45° slopes in the low-frequency region (Figure S10). Ogihara et al. (2012) reported that the projection of the 45° slope to a real axis, which is defined as R_ion_/3 (derived from transmission line model [TLM] cylindrical pores), reflects the ionic resistance of the electrolyte-filled pores inside the cathodes. The D@S−OLO cathode exhibited a lower ionic resistance (90 Ω) than the control OLO cathode (165 Ω) (Figure 3F), exhibiting the facile ion transport inside the D@S−OLO cathode. Furthermore, the Nyquist plot of the D@S−OLO cathode (Figure S10A) showed a smaller semicircle arc (an indicator of electrical contact resistance between a cathode active layer and a current collector [Landesfeind et al., 2016]) at the high-frequency region than the control OLO cathode (Figure S10B). These results demonstrate that the nanofibrous DNA@SWCNT binder plays an important role in the formation of well-developed 3D bicontinuous electron/ion conduction pathways in the D@S−OLO cathode.

To further verify the superior electron/ion transport behavior of the D@S−OLO cathode, a galvanostatic intermittent titration technique (GITT) analysis was conducted during the charging/discharging of a cell; here, a 2032–type coin cell (OLO-cathode/polyethylene separator/Li metal anode, a liquid electrolyte of 1 M LiPF_6_ in EC/DMC = 1/1 (v/v) with 0.5 wt% tris(trimethylsilyl) phosphite [TMSP] additive) was used. Figure 3G shows that the D@S−OLO cathode suppresses the increase in cell polarization upon the repeated current stimuli (applied at a current density = 1 C, with an interruption time between the pulses = 1 h), in which the obtained internal cell resistances were presented as a function of the SOC and depth of discharge (DOD) (Figure S11). In addition to the GITT analysis, the overpotential distribution of the D@S−OLO cathode in the through-thickness direction was examined using an *in situ* EIS technique (Kim et al., 2016, 2017b) (Figure S12). Figure 3H shows that the voltage difference between the top and bottom sides of the D@S−OLO cathode (ΔV ∼ 30 mV) were smaller than that in the control OLO cathode (ΔV ∼ 180 mV). Such a uniform voltage distribution in the through-thickness direction confirms the viable role of the DNA@SWCNT binder in facilitating the redox kinetics of the D@S−OLO cathode. These advantageous effects of the D@S−OLO cathode were schematically illustrated in Figure 3I, with particular attention paid to the electron/ion conduction pathways, adhesion with the Al current collector, and Mn^2+^ ion chelation.

### Cell Performance and Postmortem Analysis of the D@S−OLO Cathode

**Figure 4:**
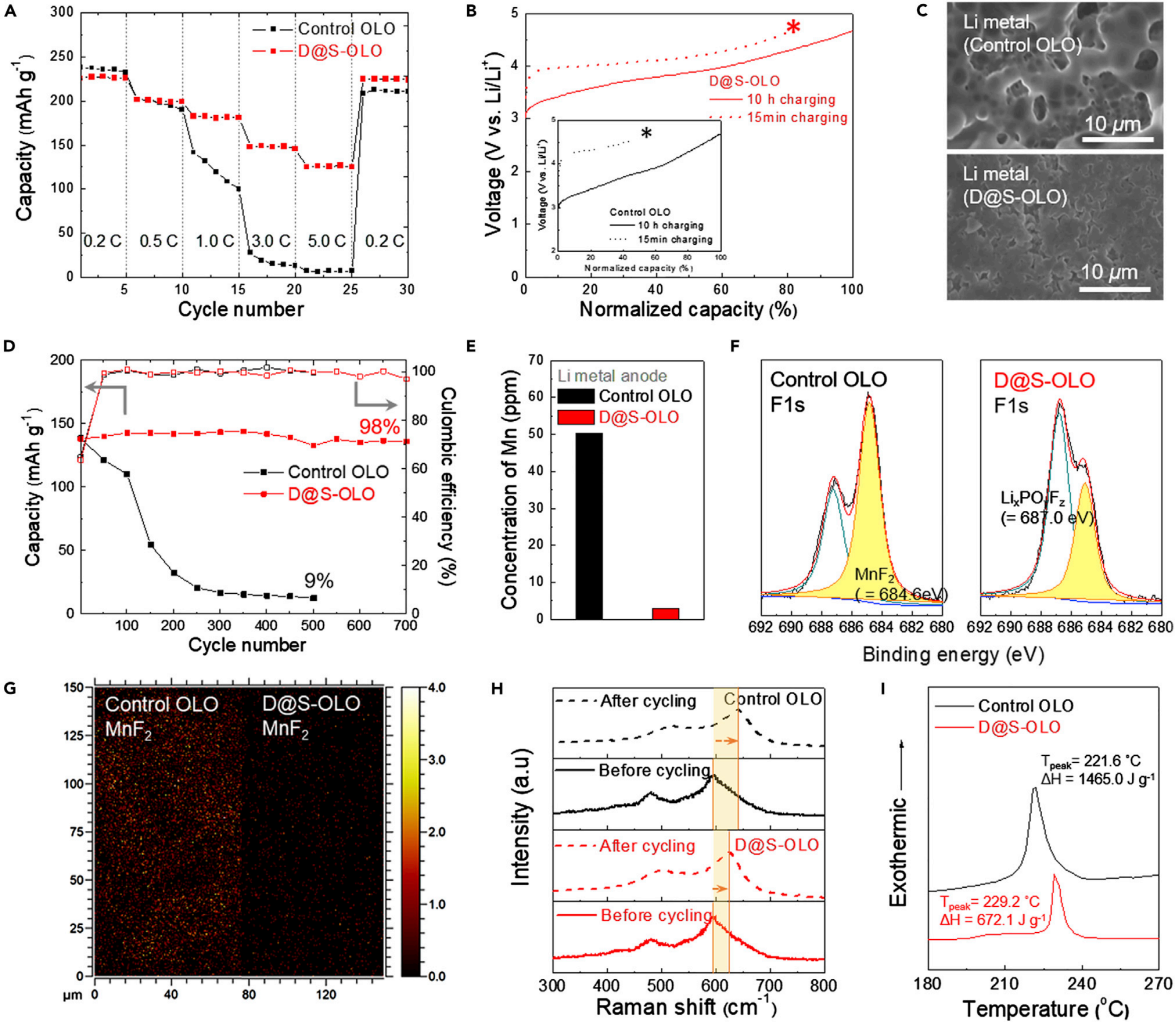
Electrochemical Superiority of the D@S−OLO Cathode (A) Discharge rate capability (D@S−OLO cathode versus control OLO cathode) over a wide range of discharge current densities (= 0.2–5.0 C) at a fixed charge current density of 0.2 C. (B) Charge profiles of the D@S−OLO cathode (versus control OLO cathode [inset]) for 15 min and 10 h. (C) Surface SEM images of the Li metal anodes (D@S−OLO cathode versus control OLO cathode) after fast (i.e., 15 min) charging (marked by the stars in Figure 4B). (D) Cycling performance (D@S−OLO cathode versus control OLO cathode) at a charge/discharge current density of 1 C/3 C under a voltage range of 2.0–4.7 V. (E–H) Postmortem analysis after 500 cycles (D@S−OLO cathode versus control OLO cathode): (E) amount of metallic Mn deposited on the Li metal anodes (estimated from ICP-MS analysis). (F) XPS F 1s spectra of the OLO cathode surface, (G) TOF-SIMS mapping images of the MnF_2_ by-products, and (H) Raman spectra of the OLO cathodes. (I) DSC thermograms showing the exothermic reactions occurring for the 4.7 V-charged D@S−OLO cathode (versus control OLO cathode).

Based on the above-mentioned structural/electrochemical characterization of the D@S−OLO cathode, its influence on the cell performance was investigated using 2032–type coin cells. We compared the discharge rate capabilities of the D@S−OLO and control OLO cathodes, in which the discharge current densities varied from 0.2 to 5.0 C under a constant charge current density of 0.2 C (Figure 4A). Driven by the highly developed 3D bicontinuous electron/ion transport channels, which can enhance the redox kinetics, the D@S−OLO cathode exhibited the higher discharge rate capability than the control OLO cathode.

In addition, we investigated the fast-charging behavior of the D@S−OLO cathode (Figure 4B). The charging capability (e.g., the relative capacity ratio after 15 min [versus 10 h]) of the D@S−OLO cathode (83%) was higher than that of the control OLO cathode (50%). Note that the value of 83% surpassed the goal (80%) of long-range EV batteries announced by the US Advanced Battery Consortium (USABC). After fast charging (i.e., 15 min) (marked by the stars in Figure 4B), the surface morphology of the Li metal anodes was analyzed (Figure 4C). The Li metal anode (coupled with the D@S−OLO cathode) had a smooth and uniform deposition surface, in contrast to the Li metal anode (assembled with the control OLO cathode), which had an uneven and irregular morphology. The stable deposition of Li on the Li metal anode is good evidence confirming the fast charging performance of the D@S−OLO cathode.

We investigated the effect of the D@S−OLO cathode on the cycling performance (Figure 4D). The D@S−OLO cathode showed an exceptionally higher capacity retention (98% after 700 cycles performed at a charge/discharge current density of 1 C/3 C) than the control OLO cathode (9% after 500 cycles). The cycling stability of the D@S–OLO cathode was verified by the small increase in the cell polarization and impedance after 500 cycles (Figure S13 and S14). Notably, the cycling performance of the D@S–OLO cathode far exceeds those of previously reported cathodes containing alternative binders/conductive agents (Table S1); the D@S–OLO cathode was compared with different cathode active materials because only a few studies reported OLO cathodes.

To better understand this superior cyclability, we conducted a postmortem analysis on the electrodes after 500 cycles. The electrodes kept their physical appearance without any powdering and shedding even after getting from disassembled cells. The ICP-MS analysis (Figure 4E) showed that the amount of metallic Mn (3 ppm) deposited on the Li metal anode (coupled with the D@S−OLO cathode) was significantly lower than that (50 ppm) of the control system. In addition, we examined the structural change of the OLO cathodes after 500 cycles. The D@S–OLO cathode was relatively clean and less contaminated compared with the control OLO cathode (Figure S15). The OLO cathode surface was further characterized by the X-ray photoelectron spectroscopy (XPS) F1s spectra (Figure 4F). For the control OLO cathode spectrum, the peak (684.6 eV) assigned to MnF_2_ was stronger than the peak (687.0 eV) ascribed to Li_x_PO_y_F_z_-containing compounds (Kim et al., 2015). The opposite tendency was observed from the D@S−OLO cathode spectrum. The MnF_2_ by-products, which are formed by unwanted side reactions occurring between Mn^2+^ ions (dissolved from cathode materials) and hydrofluoric acid (HF, generated by PF_6_^-^ and residual water in electrolytes), are known as one of critical causes for cycling decay (Han et al., 2019). The time-of-flight secondary ion mass-spectroscopy (TOF-SIMS) mapping images and intensity (Figures 4G and S16) showed that the MnF_2_ by-products were sparsely formed over a wide area, and their absolute amount was lower at the D@S−OLO cathode. The structural change of the cycled OLO active materials was examined using Raman spectroscopy (Figure 4H). After 500 cycles, the spectrum of the control OLO cathode showed the formation of a new OLO peak at 638 cm^−1^, which corresponds to a spinel-like phase (Pham et al., 2018), revealing that the structure changed during cycling. In contrast, the shift in the OLO peak was not so noticeable at the D@S–OLO cathode. These results demonstrate the advantageous role of the DNA@SWCNT binder in suppressing Mn^2+^ dissolution (conceptually illustrated in Figure 3I), eventually contributing to a better cycling performance.

We examined the thermal stability of the D@S−OLO cathode. Figure 4I shows the differential scanning calorimetry (DSC) thermograms of the OLO cathodes (charged to 4.7 V at a charge current density of 0.1 C): the D@S−OLO cathode (exothermic peak temperature = 229.2°C, exothermic heat = 672.1 J g^−1^) versus the control OLO cathode (exothermic peak temperature = 221.6°C, exothermic heat = 1465.0 J g^−1^). This DSC result shows that the DNA@SWCNT binder beneficially affects the structural stability of the OLO materials upon exposure to high-temperature conditions.

In summary, we presented a nanofibrous conductive binder based on DNA@SWCNT (i.e., DNA-wrapped SWCNT) for high-performance lithium battery electrodes. The spatially rearranged hydrophilic phosphate groups of the DNA molecules in the DNA@SWCNT binder enabled the strong adhesion with the Al current collectors and the chelation of heavy metal ions (e.g*.*, Mn^2+^), along with the introduction of the aqueous solution-based cathode sheet fabrication process. In addition, the nanofibrous feature of the DNA@SWCNT binders allowed the liquid electrolytes to easily access the OLO. Consequently, well-developed 3D bicontinuous electron/ion transport pathways were formed in the D@S−OLO cathode. Driven by these chemical functionalities and the structural uniqueness of the DNA@SWCNT binder, the resulting D@S−OLO cathode showed exceptional improvements in its fast charging capability (the relative capacity ratio after 15 min [versus 10 h] charging = 83%), cycling performance (the capacity retention = 98% after 700 cycles performed at charge/discharge current densities of 1 C/3 C), and thermal stability, which are difficult to achieve with conventional OLO cathodes (containing PVdF binders and carbon black additives). The DNA@SWCNT-based nanofibrous conductive binder strategy suggested herein provides a new concept for designing the chemistry/architecture of electrode sheets and holds great promise as a versatile/scalable platform technology for next-generation high-capacity/high-voltage electrode active materials, which often fail to reach satisfactory cell performances in conventional electrode sheets.

### Limitations of the study

The DNA@SWCNT was presented as a nanofibrous multifunctional conductive binder for high-performance LIB electrodes. To enable versatile and practical uses of the nanofibrous conductive binder concept proposed herein, future studies will be focused on exploring applications to newly emerging high-capacity/high-voltage electrode active materials and developing cost-competitive alternative materials that can replace DNA and SWCNT.

### Lead Contact

Futher information and requests for resoursesand reagents should be directed to and will be fulfilled by the Lead Contact, Sang-Young Lee (sangyounglee87@gmail.com).

### Materials Availability

This study did not generate new unique reagents.

### Data and Code Availability

This study did not generate/analyze [datasets/codes].

## Methods

All methods can be found in the accompanying Transparent Methods supplemental file.

## Supplemental Information

Document S1. Transparent Methods, Figures S1–S16, and Table S1
