# Supplementary material for: Nanofibrous Conductive Binders Based on DNA-Wrapped Carbon Nanotubes for Lithium Battery Electrodes
Source: iScience. 2020 Oct 28;23(11):101739. doi: 10.1016/j.isci.2020.101739 (PMC7670196; doi:10.1016/j.isci.2020.101739)
Supplement: Document S1. Transparent Methods, Figures S1–S16, and Table S1 [file mmc1.pdf]

iScience, Volume *23*

Supplemental Information

Nanofibrous Conductive Binders Based on DNA-Wrapped Carbon Nanotubes for Lithium Battery Electrodes

Ju-Myung Kim, Seung-Hyeok Kim, Nag Young Kim, Myeong-Hwa Ryou, Hongyeul Bae, Jin Hong Kim, Young-Gi Lee, and Sang-Young Lee

Supporting Information

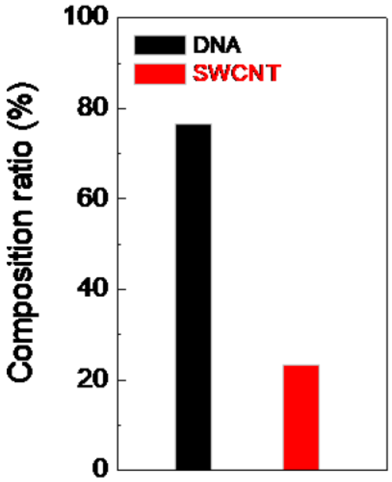

Figure S1. Composition ratio of DNA and SWCNT in the DNA@SWCNT (estimated by elemental analysis), Related to Figure 1.

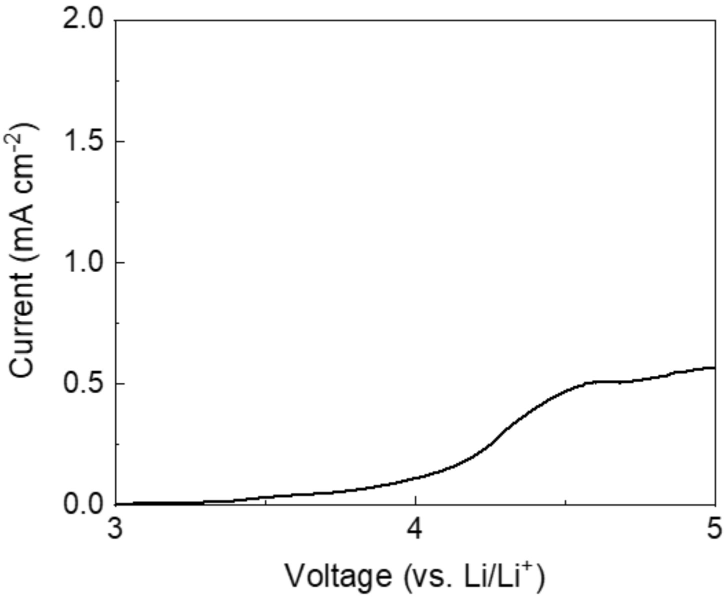

Figure S2. Electrochemical stability window of the control film (PVDF/carbon black powder = 5/5 (w/w)), Related to Figure 2.

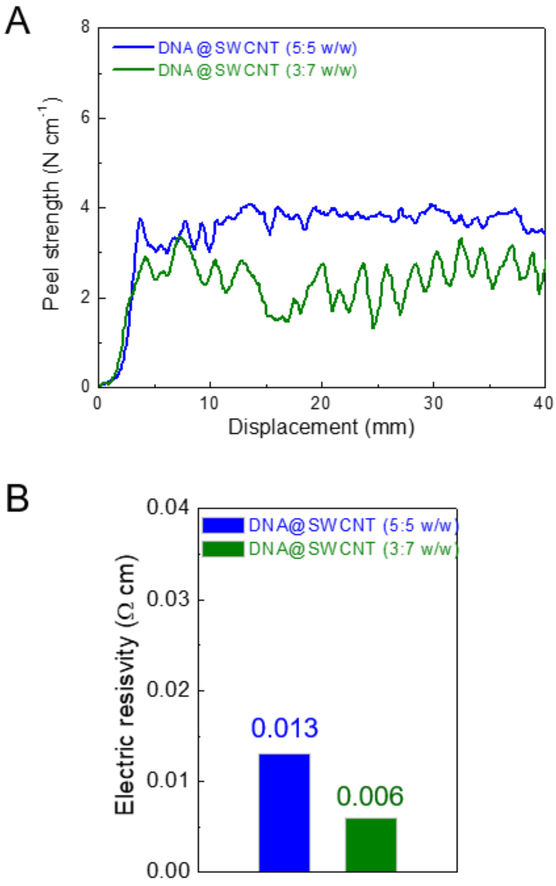

Figure S3. (A) 180° peel-off test, showing the adhesion between the DNA@SWCNT films (initial composition ratios of the DNA/SWCNT = 3/7 and 5/5 (w/w)) and Al current collector. (B) Electric resistivity of the DNA@SWCNT films (initial composition ratios of the DNA/SWCNT = 3/7 and 5/5 (w/w)), Related to Figure 2.

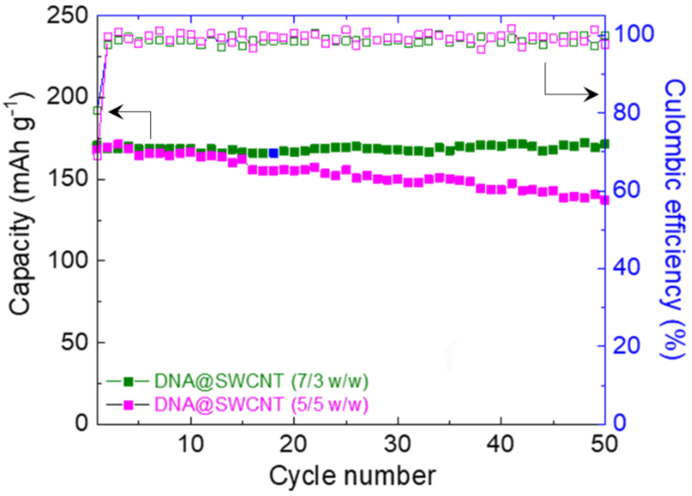

Figure S4. Cycling performance (at a charge/discharge current density of 1C/1C) of the OLO cathodes with different composition ratios (DNA/SWCNT = 7/3 vs. 5/5 (w/w)), Related to Figure 2.

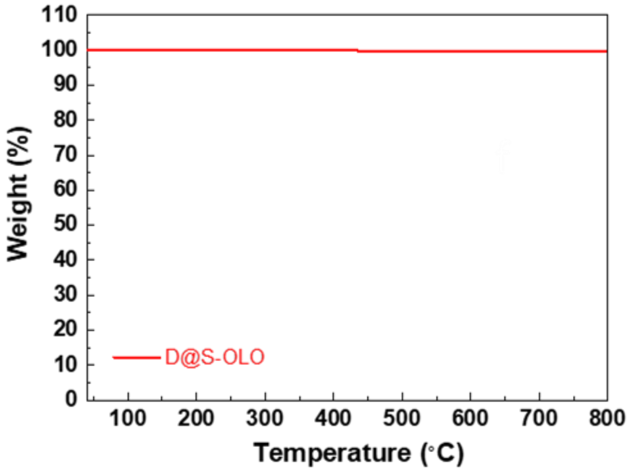

Figure S5. TGA profile of the D@S–OLO cathode, Related to Figure 3.

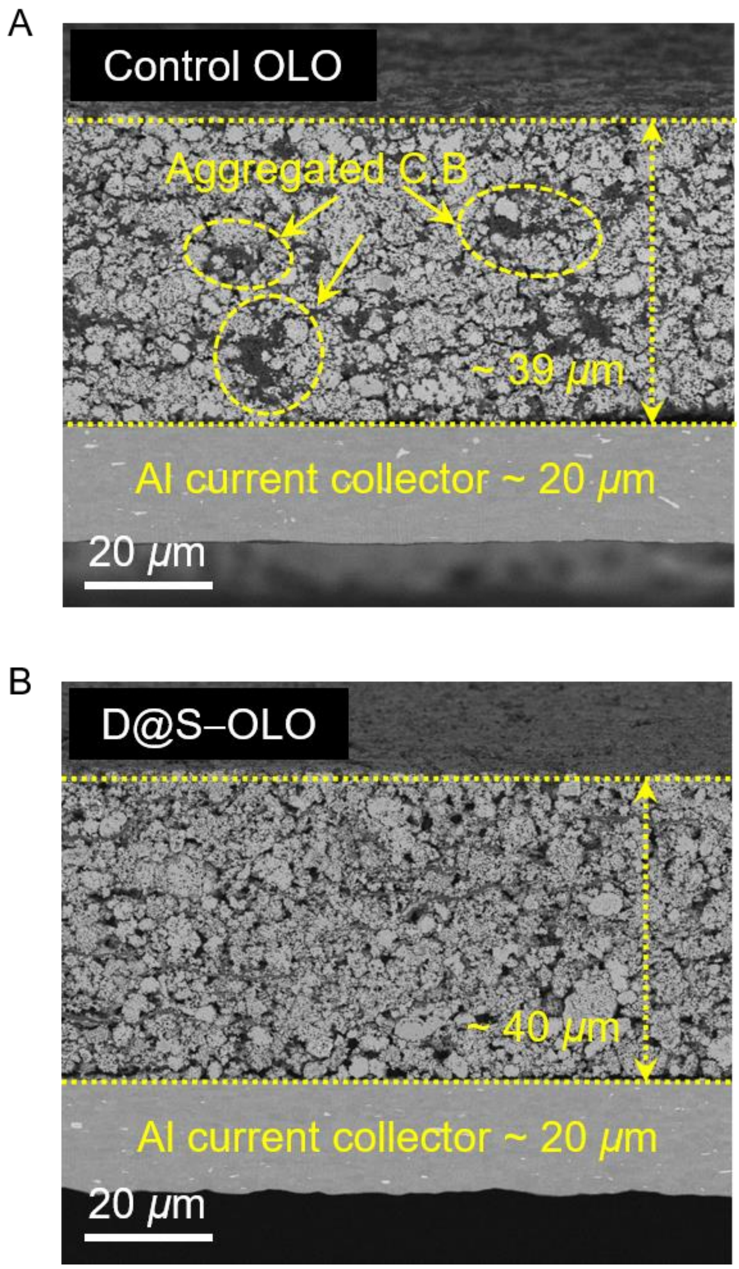

Figure S6. Cross-sectional SEM images of (A) control OLO cathode and (B) D@S−OLO cathode, Related to Figure 3.

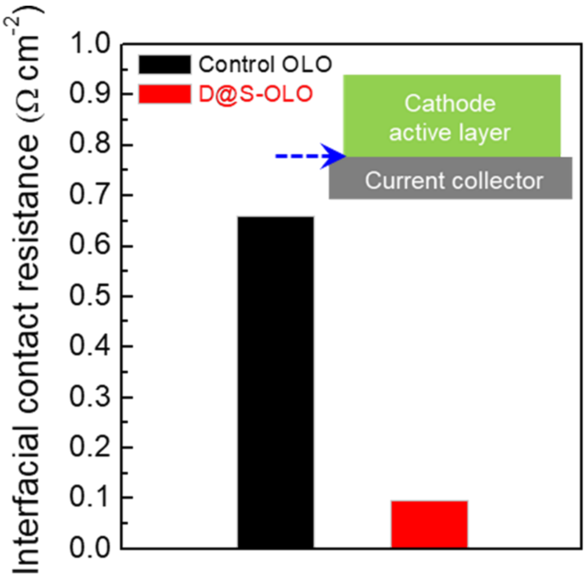

Figure S7. Interfacial electrical resistance between the cathode active layers and Al current collectors: D@S–OLO cathode vs. control OLO cathode, Related to Figure 3.

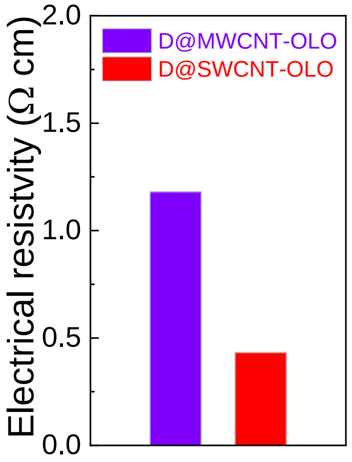

Figure S8. Comparison of electrical resistivity between DNA@MWCNT−OLO cathode and DNA@SWCNT−OLO (D@S−OLO) cathode, Related to Figure 3.

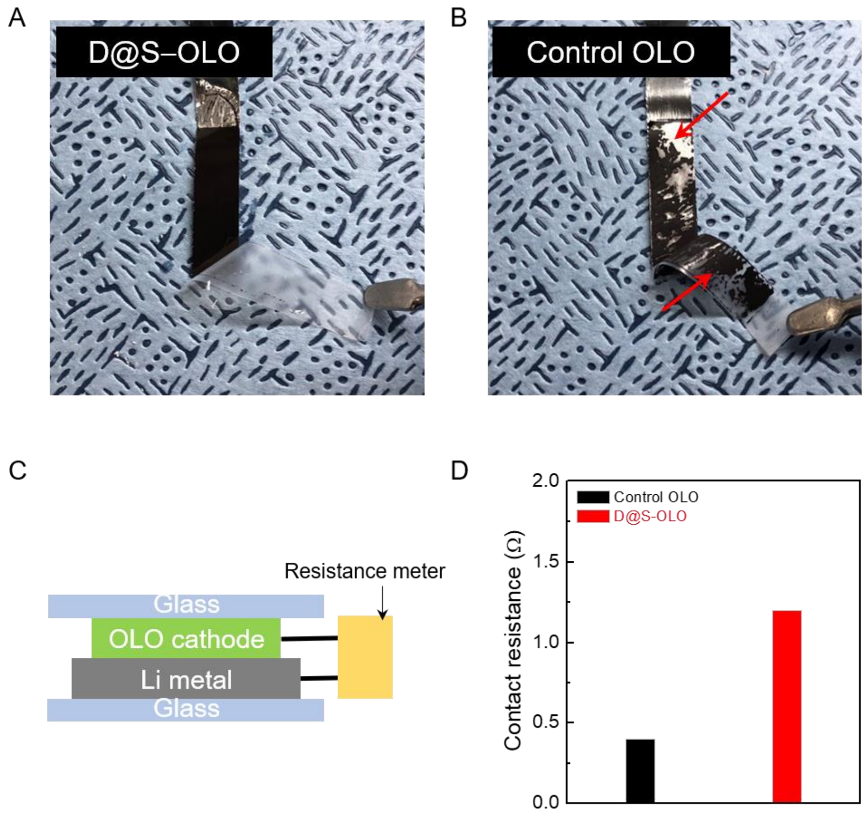

Figure S9. Photographs after the 180° peel-off test (after being soaked in the liquid electrolyte for 5 min) was performed for the (A) D@S–OLO cathode and (B) control OLO cathode. (C) Schematic illustration depicting a model system designed for the measurement of the contact resistance between the OLO cathodes (taken after the peel-off test) and Li metals. (D) Contact resistance between the OLO cathodes (taken after the peel-off test) and Li metals: D@S–OLO cathode vs. control OLO cathode, Related to Figure 3.

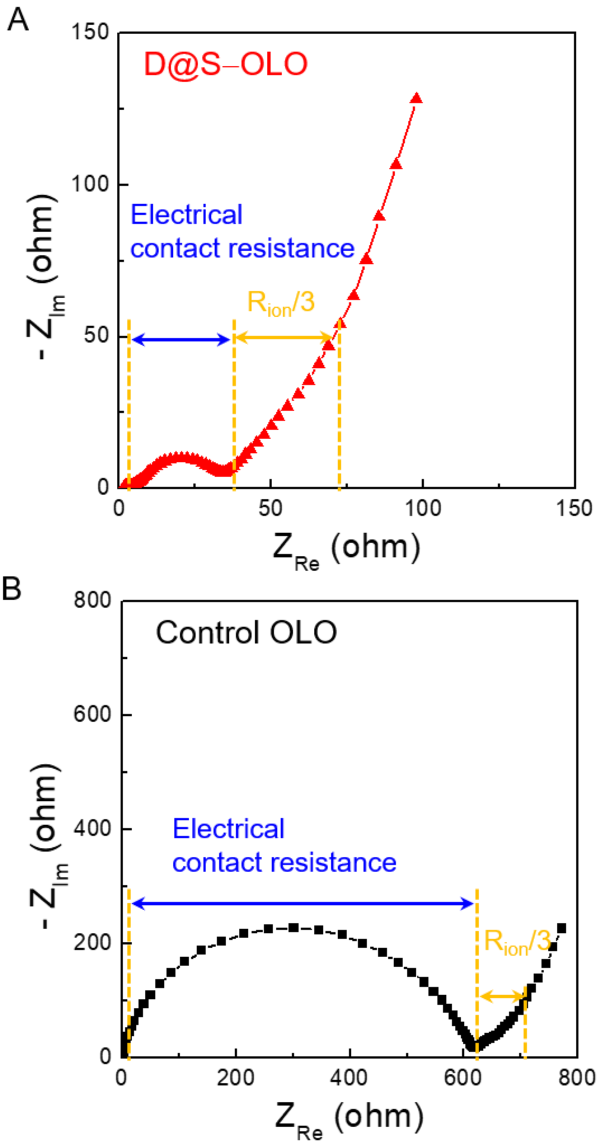

Figure S10. Nyquist plots of the symmetric cells (comprising two identical cathodes at 50% SOC): (A) D@S–OLO cathode and (B) control OLO cathode, Related to Figure 3.

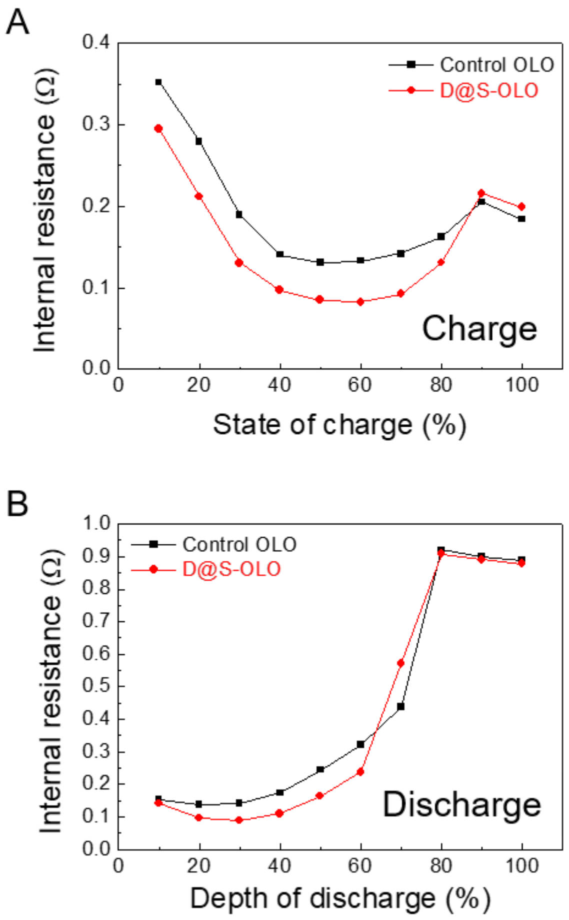

Figure S11. Variation in the internal cell resistance (estimated from the GITT profiles shown in Figure 3G) as a function of: the (A) SOC and (B) DOD, Related to Figure 3.

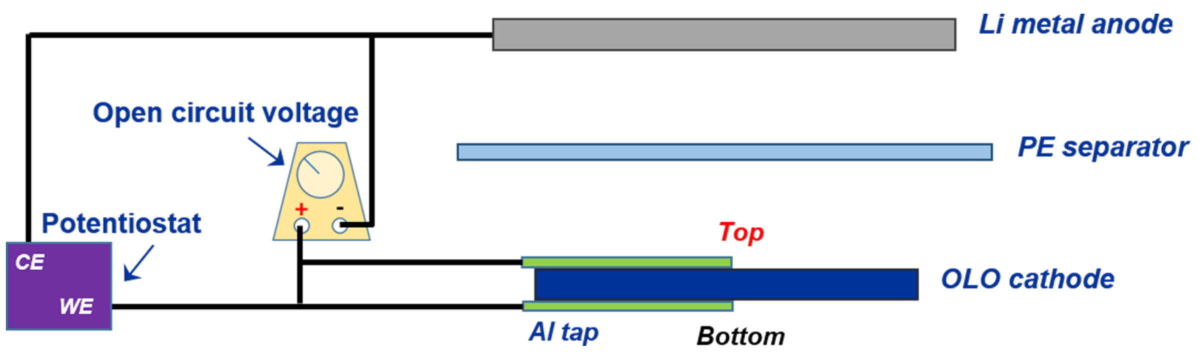

Figure S12. Schematic representation depicting a cell designed for the *in situ* EIS measurement of the thickness-directional overpotential distribution (*i.e.*, voltage difference between the top and bottom side) of the OLO cathodes, Related to Figure 3.

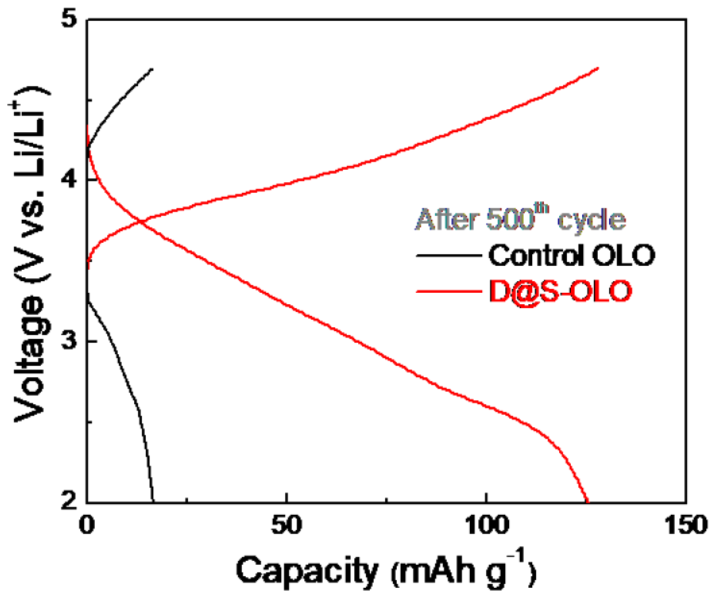

Figure S13. Charge/discharge profiles (D@S–OLO cathode vs. control OLO cathode) obtained after 500 cycles, Related to Figure 4.

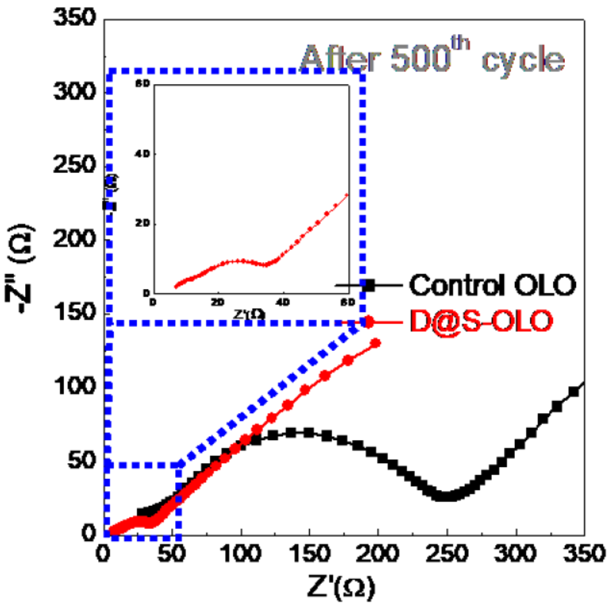

Figure S14. EIS spectra (D@S–OLO cathode vs. control OLO cathode) obtained after 500 cycles, Related to Figure 4.

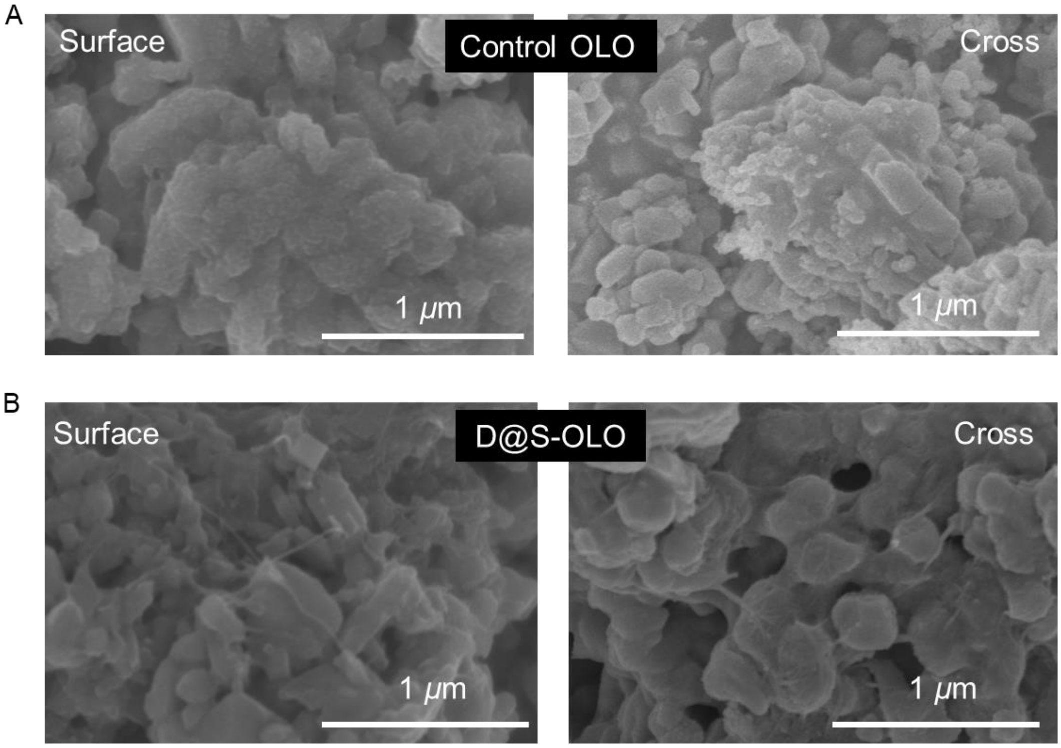

Figure S15. Surface and cross-sectional SEM images (after 500 cycles) of (A) control OLO cathode and (B) D@S−OLO cathode, Related to Figure 4.

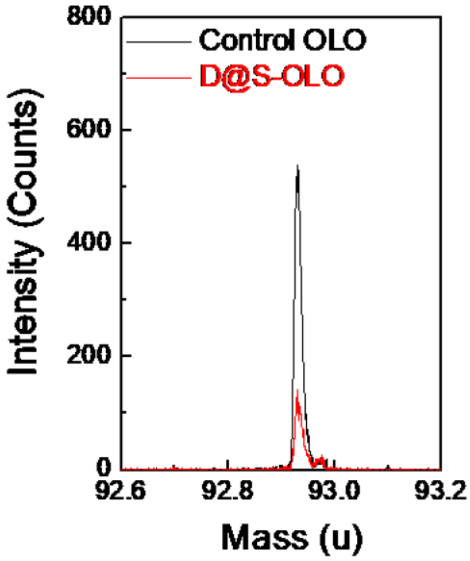

Figure S16. TOF-SIMS intensities of the MnF_2 byproducts on the D@S−OLO cathode (vs. control OLO cathode) after 500 cycles, Related to Figure 4.

Cathode active material	Binder/ Conductive agent	Cycle number	Charge/discharge current density [C/C]	Cycle retention [%]	Content of active materials [wt.%]	Ref.
OLO	DNA@SWCNT	700	1.0/3.0	98.0	92	This study
	Xanthan Gum/C.B	100	0.5/0.5	98.4	80	17
	Fluorinated polyimide/C.B	50	0.2/0.2	94.0	75	37
$LiFePO_4$	PPy hydrogel	500	1.0/1.0	75.0	85	11
	SA-PProDOT	400	1.0/1.0	86.6	80	12
FeF_2	$Ni@Ai_2O_3$	100	0.5/0.5	-	-	13
$LNi_{0.5}Mn_{1.5}O_4$	LiPAA/C.B	80	0.5/0.5	90.0	90	14
$Li_3V_2(PO_4)_3$		300	1.0/1.0	97.4	75	15
$LiCoO_2$	SBR-CMC/A.B	50	0.1/0.1	95.0	80	16
$LiNi_{1/3}Co_{1/3}Mn_{1/3}O_2$	Polyacrylic latex/C.B	200	0.2/0.2	92.0	90	18

Table S1. Comparison of the electrochemical performances of the D@S−OLO cathode (this study) and previously reported cathodes containing alternative binders/conductive agents, Related to Figure 4.

Transparent Methods

Synthesis of the DNA@SWCNT

To synthesize the DNA@SWCNT, deoxyribonucleic acid (DNA) sodium salts (from salmon testes, Aldrich) were mixed with single-walled carbon nanotubes (SWCNT, Tuball) at an initial composition ratio of DNA/SWCNT = 7/3 (w/w) in an aqueous solution. Prior to mixing, the SWCNT were purified by refluxing in 3 M nitric acid at 120°C for 16 h.(Hu et al., 2003) The mixture suspension was centrifuged (Combi-514R, Hanil Science Medical) for 90 min at 16,000 g to eliminate the insoluble components. The resulting suspension was subjected to freeze-drying, thus yielding the DNA@SWCNT powders.

Structural/physicochemical characterization of the DNA@SWCNT

To characterize the dispersion state of the DNA@SWCNT in the aqueous suspension, we conducted dynamic light scattering (DLS, Zetasizer Nano ZS, Malvern) and transmission electron microscopy (TEM, JEM-2100F, JEOL) analyses. The composition ratio of the DNA/SWCNT in the DNA@SWCNT was estimated by an elemental analyzer (EA, Flash 2000, Thermo). The electrical resistivities were measured using a four-point probe technique (CMT-SR1000N, Advanced Instrument Tech). The adhesion strength between the electrode active layers and the Al current collectors was measured by a universal testing machine (DA-01, Petrol LAB) at a peel speed of 300 mm min^{-1}. To quantitatively estimate the Mn^{2+} chelation ability of the DNA@SWCNT, manganese perchlorate solution (10 mM $Mn(ClO_4)_2$-containing 1.0 M $LiPF_6$ in EC/DMC = 1/1 (v/v)) was prepared as a model solution. After being soaked in the solution for 2 h at room temperature, the samples were washed with DMC solvents, and then, the amount of the captured Mn^{2+} ions was estimated by ICP-MS (ELAN DRC-II, Perkin Elmer) analysis. The electrochemical stability window of the DNA@SWCNT was investigated using linear sweep voltammetry (LSV) performed on a working electrode composed of stainless-steel

and a counter/reference electrode composed of Li metal, and the LSV measurements were performed with a scan rate of 1.0 mV s^{-1}.

Electrochemical characterization of the OLO cathodes

The D@S–OLO cathode was fabricated by casting a water-based slurry mixture of 92 wt% OLO (HE 5050, Toda) and 8 wt% DNA@SWCNT binders on an Al current collector. The mass loading of OLO in the D@S–OLO cathode was = 7 mg cm^{-2}. The control OLO cathode, which consisted of a 92 wt% OLO and 8 wt% PVdF/carbon black (1/1 w/w) mixture, was fabricated by casting the NMP-based slurry on an Al current collector. The electrochemical performance of the OLO cathodes was characterized using 2032-type coin cells (= OLO cathode/polyethylene separator (thickness ~ 20 μm, Toray–Tonen)/Li metal anode). A liquid electrolyte of 1 M $LiPF_6$ in EC/DMC = 1/1 (v/v) with a 0.5 wt% TMSP additive was used. The cell was assembled in an argon-filled glove box. The cell performance was investigated using a cycle tester (PNE Solution) under various charge/discharge conditions. The GITT profiles and AC impedance of the cells were obtained using a potentiostat/galvanostat (VSP classic, Bio–Logic). To determine R_{ion} from the electrochemical impedance spectroscopy (EIS) measurement, two identical cathodes were assembled in a symmetric cell with a state of charge (SOC) of 50%.

Structural/physicochemical characterization of the OLO cathodes

The surface and cross-sectional morphologies of the OLO cathodes were investigated using field emission secondary electron microscopy (FE-SEM, S-4800, Hitachi) and energy-dispersive X-ray spectroscopy (EDS, JSM 6400, JEOL). The electrical resistivity and contact resistance of the OLO cathodes were measured using an electrode measurement system (RM2610, Hioki). The inductively coupled plasma-mass spectrometry (ICP-MS, ELAN DRC–II, Perkin Elmer) analysis was conducted to quantitatively estimate the Mn deposited on the Li

metal anode after the cycling test. The interfacial exothermic reaction occurring between the charged OLO and liquid electrolyte was examined using differential scanning calorimetry (DSC, Q200, TA), in which the cells were charged to 4.7 V at a current density of 0.1 C and then disassembled in a glove box to obtain the charged cathode. The Raman spectra were recorded by a micro-Raman spectrometer (alpha 300R, WITec) equipped with a He/Ne laser (632 nm). The chemical change of the cathode surface after the cycling test was analyzed by using TOF-SIMS (ION TOF) with a Bi_3^{2+} gun (25 keV, 1 pA).

Reference

Hu, H., Zhao, B., Itkis, M. E., Haddon, R. C. (2003). Nitric acid purification of single-walled carbon nanotubes. J. Phys. Chem. B 107, 13838-13842.
